# Supplementary material for: The pht4;1-3 mutant line contains a loss of function allele in the Fatty Acid Desaturase 7 gene caused by a remnant inactivated selection marker—a cautionary tale
Source: PeerJ. 2017 Dec 1;5:e4134. doi: 10.7717/peerj.4134 (PMC5713625; doi:10.7717/peerj.4134)
Supplement: Data S1 — Letters marked in blue indicate IAAH stop codon and letters in yellow indicate FAD7 genomic sequence. Genomic sequence of FAD7 from wild type Col-0 (TAIR12, http://www.arabidopsis.org). Start and stop codon are highlighted in blue. Capital letters indicate exon and lower case letters indicate introns. Yellow letters indicate sequence identified in the pht4;1-3 mutant (see sequence above). [file peerj-05-4134-s001.docx]

Supplemental data 1. DNA sequence of insert located in *FAD7* in the *pht4;1-*3 mutant line. Letters marked in blue indicate IAAH stop codon and letters in yellow indicate *FAD7* genomic sequence.

GTCAGCATCATCACACCAAAAGTTAGGCCCGAATAGTTTGAAATTAGAAAGCTCGCAATTGAGGTCTACAGGCCAAATTCGCTCTTAGCCGTACAATATTACTCACCGGTGCGATGCCCCCCATCGTAGGTGAAGGTGGAAATTAATGATCCATCTTGaGACCACAGGCCCACAACAGCTACCAGTTTCCTCAAGGGTCCACCAAAAACGTAAGCGCTTACGTACATGGTCGaTAAGAAAAGGCAATTTGTAGATGATcgCCATGGAAACGCCGGAGTAGgtCTTTGCGGCATTCCaCTCTGTTTTAaGGcGAaCATCGCTACCGGCGTATTTCCCaCAAGCGCCGCTACGCCGGCGCTGATAAACCACTTGCCAAAGATACCATCCCGCGTCGCAGAAAGACTTTTTTCAGCTGGAGCACTGCCGGGTGCCTCGGGAAATATGCATGAGTTATCGTTTGGAATTACAAGCAACAACTATGCCACCGGGGCGGTGCGAAACCCGTGGAATCCAGATCTGATACCAGGGGGCTCAAGCGGTGGTGTGGCTGCTGCGGTAGCAAGCCGATTGATGTTAGGCGGCATAGGCACCGATACCGGTGCATCTGTTCGCCTACCCGCAGCCCTGTGTGGCGTAGTAGGATTTCGACCGACGCTTGGTAGATATCCGGGAGATCGGATAATACCGGTTAGCCCTACCCGGGACACTCCCGGAATCATAGCGCAGTGCGTAGCCGATGTTGTAATCCTCGACCGGATAATTTCCGGCACACCGGAGAGAATACCACCCGTGCCGCTGAAGGGGCTAAGGATCGGCCTCCCTACAACCTACTTTTATGATGACCTTGATGCTGATGTGGCCCTAGCAGCTGAAACAACGATTCGCCTGCTAgCAAACAAAGGCGTAACTTTTGTTGAAGCTAACATTCCCCACCTTGACGAACTGAATAAAGGGGCCAGCTTCCCAGTTGCACTCTATGAAtTTCCACACGCTCTAAAAACAGTATCTCGACGACTTTGtAAAAACTGtTTCTTTTTCTGACGTCaTCAAAgGAATTCgtAnCcntGATGTAnCCAACatTtGCCAAtGCGCAAaTtGATGgacATCaaAtTtCCAAngCTGAATATGAACTGGCCCGCCACTCCTTCAGACCAAGACTTCAAGCCACCTATCGCAACTACTTCAAACTGAATAGATTAGATGCTATTCTCTTCCCAACAGCACCCTTGGTGGCCAGACCCATAGGTCAGGATTCCTCAGTTATCCACAATGGCACGATGCTGGACACATTCAAGATCTACGTGCGAAATGTGGACCCAAGCAGCAACGCAGGCCTACCTGGCTTGAGCATTCCTGTTTGCCTGACACCTGATCGCTTGCCTGTTGGAATGGAGATCGATGGATTAGCGGATTCAGACCAACGTCTGTTAGCAATCGGGGGGGCATTGGAAGAAGCCATTGGATTCCGATATTTTGCCGGTTTACCCAATTAAACTTTCTACCATGTTCGTTTTTACAATTTTTCAGATTGATGACAATCAATCCTTGTATTGCGTCTATGAACAACAGTGCCTTATGTTATAAATCGAATAATAACTTGCGATGGAGATTTTGAACAAACTTTAATTTATGATTTAACCAATAAAAGTCTTTGCAATAATCATGTTCGATAAATAATTTTATTATCAGTGATAAACTTTAGATATTTCTTGGAATAGGCATCTTCATATAACAATAATTCTTTTTCGAATTTAAATTATATATCTTAAGCAAATTACATTTTTCCTAAATTATAGGGAAATATAATTAAAGTTCAAGCAATCCTATTTAGGCATAAAGTAGTTAATACAATTTATATAATATATAATTTGCCGTCCACTATCCTTTACAAATTTGTCAAATCTGCATTATGAAATTAATGACTTTTTTGAATTGTCTCGCCGTGTGAGGACAGACGTGAAGGCACCCACCTTTTAATTTGGATGCACCTCTCACCTGCTACAACATCGATACCGTCGATGGGTGGATTTATCACAAATGGGACCCGCCGCCGACAGAGGTGTGATGTTAGGCCAGGACTTTGAAAATTTGCGCAACTATCGTATAGTGGCCGACAAATTGACGCCGAGTTGACAGACTGCCTAGCATTTGAGTGAATTATGTAAGGTAATGGGCTACACTGAATTGGTAGCTCAAACTGTCAGTATTTATGTATATGAGTGTATATTTTCGCATAATCTCAGACCAATCTGAAGATGAAATGGGTATCTGGGAATGGCGAAATCAAGGCATCGATCGTGAAGTTTCTCATCTAAGCCCCCATTTGGACGTGAATGTAGACACGTCGAAATAAAGATTTCCGAATTAGAATAATTTGTTTATTGCTTTCGCCTATAAATACGACGGATCGTAATTTGTcgTTTTATCAAAATGTACTTTCATTTTATAATAACGCTGCGGACATCTACATTTTTGAATTGAAAAAAAaTTGGTAATTACTCTTTCTTTTTCTCCATATTGACCATCATACTCATTGCTGATCCATGTAGATTTCCCGGACATGAAGCCATTTACAATTGAATATATCCTGGTGAAAAGTGAAAAGTCTTATCAGAATGTGGTATACGACCTCTCCCCAGAATCTACACAACACCCAGATCCAATTTCCTCTCCAACAACAACAAATTCAGACCATCACTTTCTTCTTCTTCTTACAAAACATCATCATCTCCTCTGTCTTTTGGTCTGAATTCACGAGATGGGTTCACGAGGAATTGGGCGTTGAATGTGAGCACACCATTAACGACACCAATATTTGAGGAGTCTCCATTGGAGGAAGATAAT

Genomic sequence of *FAD7* from wild type Col-0 (TAIR12, www.arabidopsis.org). Start and stop codon are highlighted in blue. Capital letters indicate exon and lower case letters indicate introns. Yellow letters indicate sequence identified in the pht4;1-3 mutant (see sequence above).

ATGGCGAACTTGGTCTTATCAGAATGTGGTATACGACCTCTCCCCAGAATCTACACAACACCCAGATCCAATTTCCTCTCCAACAACAACAAATTCAGACCATCACTTTCTTCTTCTTCTTACAAAACATCATCATCTCCTCTGTCTTTTGGTCTGAATTCACGAGATGGGTTCACGAGGAATTGGGCGTTGAATGTGAGCACACCATTAACGACACCAATATTTGAGGAGTCTCCATTGGAGGAAGATAATAAACAGAGATTCGATCCAGGTGCGCCTCCTCCGTTCAATTTAGCTGATATTAGAGCAGCTATACCTAAGCATTGTTGGGTTAAGAATCCATGGAAGTCTTTGAGTTATGTCGTCAGAGACGTCGCTATCGTCTTTGCATTGGCTGCTGGAGCTGCTTACCTCAACAATTGGATTGTTTGGCCTCTCTATTGGCTCGCTCAAGGAACCATGTTTTGGGCTCTCTTTGTTCTTGGTCATGACTGgtaaacttaaaaaccctaacttttttcttgttttctcctctgctttagtctcctttagcctttgatttggtcaactttggatgattccaaagaaccaatcgaacaaattggtctttatccatatctcttcaaatagctttaggacataattggtctctcaggtaacaagctgtcattatcatcatactcatcatgttgctagtagaccaacccaattggcaactgtttgttggttttgcaactgtgtaatctgctttgaattgtgaacaaaattattgatttatgttgattacattgcagTGGACATGGTAGTTTCTCAAATGATCCGAAGTTGAACAGTGTGGTCGGTCATCTTCTTCATTCCTCAATTCTGGTCCCATACCATGGCTGgtgagttttgctttcagaccattcttctctaaaaccacttgcagaatctcatcttcttcatgtaaaaatatgactttgcagGAGAATTAGTCACAGAACTCACCACCAGAACCATGGACATGTTGAGAATGACGAATCTTGGCATCCTgtaagtcaaaaacgtatttttttggttatcttgttttagtcctgtggtgtttcttagatgcagttttattaactgtttctgtaactgcagATGTCTGAGAAAATCTACAATACTTTGGACAAGCCGACTAGATTCTTTAGATTTACACTGCCTCTCGTGATGCTTGCATACCCTTTCTACTTGgtaagaactcctctatttgttatggtaacttaagctgccacaccaagtaaaaaagctcatgtctattcttctgtttcagTGGGCTCGAAGTCCGGGGAAAAAGGGTTCTCATTACCATCCAGACAGTGACTTGTTCCTCCCTAAAGAGAGAAAGGATGTCCTCACTTCTACTGCTTGTTGGACTGCAATGGCTGCTCTGCTTGTTTGTCTCAACTTCACAATCGGTCCAATTCAAATGCTCAAACTTTATGGAATTCCTTACTGGgtaatgcgccgctgttactcccctgtttcagcctgagcaatttgtgtattatttcctctgccttactcaaaaaggtttttatgtcaaatacagATAAATGTAATGTGGTTGGACTTTGTGACTTACCTGCATCACCATGGTCATGAAGATAAGCTTCCTTGGTACCGTGGCAAGgtaaaatacatattctctgcttccactgttctttgactacatcgctctttcttttaaggttaaagccaactggtgtgtaaatctcatgattctcccaaaacagGAGTGGAGTTACCTGAGAGGAGGACTTACAACATTGGATCGTGACTACGGATTGATCAATAACATCCATCATGATATTGGAACTCATGTGATACATCATCTTTTCCCGCAGATCCCACATTATCATCTAGTAGAAGCAgtaagtaaattgaaagtaaagactgtttgtgtttttggtgttcatgctagtttccctgactcttgctccactgttatgcagACAGAAGCAGCTAAACCAGTATTAGGGAAGTATTACAGGGAGCCTGATAAGTCTGGACCGTTGCCATTACATTTACTGGAAATTCTAGCGAAAAGTATAAAAGAAGATCATTACGTGAGCGACGAAGGAGAAGTTGTATACTATAAAGCAGATCCAAATCTCTATGGAGAGGTCAAAGTAAGAGCAGATTGA
